# Adolescents’ knowledge and perceptions of sexual and reproductive health rights and access to services in the Greater Banjul Area, the Gambia

**DOI:** 10.11604/pamj.2025.51.2.47467

**Published:** 2025-05-05

**Authors:** Binta Jallow, Olutosin Alaba Awolude, Haddy Tunkara Bah, Million Teklay Solomon, Iacane Bampoque, Ines Nshimirimana

**Affiliations:** 1 Pan African University Life and Earth Sciences Institute (including Health and Agriculture), Ibadan, Nigeria,; 2 Department of Obstetrics and Gynaecology, College of Medicine, University of Ibadan, Ibadan, Nigeria,; 3 Department of Nursing and Reproductive Health, University of The Gambia, Serrekunda, Gambia

**Keywords:** Adolescents, sexual and reproductive health (SRH), sexual and reproductive health education, Gambia, health-seeking behaviours

## Abstract

**Introduction:**

adolescents in the Gambia face significant challenges in accessing sexual and reproductive health (SRH) services, hindered by sociocultural barriers, inadequate education, and systemic limitations. Despite representing a significant proportion of the population, limited research addresses the sociocultural and institutional factors shaping their SRH awareness and access. This study bridges these gaps by assessing adolescents' knowledge, perceptions, and access to SRH services while identifying the associated barriers and determinants in the Greater Banjul Area.

**Methods:**

this cross-sectional study included 424 secondary school students aged 15-19 years in grades 10 and 11 of selected private and public schools. Data were collected using structured questionnaires and analyzed with descriptive statistics, chi-square tests, and logistic regression.

**Results:**

revealed that only 34.2% of participants were aware of SRH rights, and only 12.7% had attended formal SRH training. Knowledge gaps were evident, with only 24.1% aware of contraception rights and 23.6% understanding consent rights. Barriers associated with these findings included lack of information (41.5%), cultural taboos (34.9%), and fear of judgment (22.9%). Only 22.4% accessed SRH services, mainly in public clinics (8.3%), with a satisfaction rate of 15.1%. Logistic regression showed public school students had less access (AOR = 0.320, 95% CI: 0.190-0.539, p < 0.001) and fewer barriers (AOR = 0.253, 95% CI: 0.107-0.601, p =0.002).

**Conclusion:**

this study emphasizes the need for age-appropriate SRH education, expanded youth-friendly services, and community engagement to reduce cultural barriers and improve equitable access for Gambian adolescents.

## Introduction

Adolescence is the age range between 10 and 19 years old, a stage of transition through which the development is both physiological, emotional, and social to build a life of health in the future [1,2]. It is the cornerstone upon which sexual and reproductive health significantly depends on one's long-term health outcomes. This phase involves significant brain development and increased sensitivity to environmental influences [2]. However, in a low-income country such as the Gambia, adolescents face significant challenges that hinder their access to adequate sexual and reproductive health (SRH) knowledge and interventions. Despite accounting for over 32% of the population, Gambian adolescents are disproportionately affected by high-risk behaviors, including early sexual activity, unprotected sex, substance abuse, and gender-based violence [3]. This situation is compounded by sociocultural norms, limited access to youth-friendly healthcare services, and inadequate SRH education, which leave adolescents vulnerable to poor health outcomes [4]. The prevalence of teenage pregnancy in Gambia is 13.42%, with 14% of adolescent females aged 15-19 years either pregnant or already mothers [5]. Early childbearing is very prevalent in rural settings and is almost double what is witnessed in urban settings [3]. This results in high risks of morbidity and mortality among young mothers and their neonates due to severe impacts on the maternal and neonatal health outcomes [6]. Existing cultural and religious norms further exacerbate these issues because they enforce taboos regarding SRH discussions, especially among families, leaving adolescents without the proper guidance to make well-thought-out decisions [7,8]. These barriers have consequences in the prevalence of sexually transmitted infections such as HIV. The HIV prevalence among those aged 15-24 years increased from 0.8% in 2002 to 2.4% in 2006, which underscores the need for targeted SRH interventions [9].

The complex nature of barriers to adolescents accessing SRH services includes social stigma surrounding adolescent SRH, financial constraints, and the availability of few youth-friendly services [10,11]. Cultural norms typically avoid open discussion of SRH matters with adolescents, especially girls [10]. Specific systemic barriers include lack of trained personnel in the healthcare system and poor service quality that further burdens access to services [12]. This leads to a lack of adequate information in adolescents regarding contraception, STIs, and reproductive rights, hence failing to make rational decisions on their health. Implications include higher numbers of unintended pregnancies and unsafe abortions and the persistence of risky sexual practices that further compromise their health and development [3]. Despite increasing research interest in adolescent sexual and reproductive health (SRH) over the past decade, studies in sub-Saharan Africa, including Gambia, remain limited and unevenly distributed across the region [13]. Existing research highlights low contraceptive use among adolescents, with barriers such as limited knowledge, negative provider attitudes, and cultural misconceptions [3,7]. Although the majority of research has concentrated on topics such as HIV, sexual behavior, and service accessibility, there are still substantial knowledge gaps about the broader socioeconomic and institutional elements influencing teenagers' SRH awareness and health-seeking behavior. This raises several important research questions: What is the extent of adolescents´ knowledge regarding SRH rights and services in urban Gambia? How do adolescents perceive SRH services, and what barriers influence their access to these services? What factors shape adolescents´ health-seeking behaviors regarding SRH, including the roles of stigma, cultural norms, and healthcare quality? The present study, therefore, tries to fill these critical knowledge gaps by assessing adolescents´ knowledge, perceptions, and access to sexual and reproductive health services in urban Gambia. Additionally, it investigates the factors influencing their health-seeking behaviors.

## Methods

**Study areas:** this study was carried out in the Greater Banjul Area (GBA), the most urbanized and densely populated part of Gambia. The GBA includes the capital city, Banjul, and the Kanifing Municipality. It is the major economic, administrative, and educational center in the country. According to the 2013 Population and Housing Census, the estimated population of GBA was about 413,397, representing approximately 22% of the national population. The region is characterized by well-developed infrastructure, including extended health and educational facilities; access to services is reportedly better than in rural areas. Linguistically, the GBA is diverse, with English as the official language and local languages such as Mandinka, Wolof, and Fula widely spoken. The GBA hosts a significant proportion of the country´s adolescent population, making it an ideal setting for examining sexual and reproductive health (SRH) dynamics. The region includes 43 secondary schools, of which 26 are public and 17 are private [14], catering to a large youth population and further emphasizing its relevance as a site for studying adolescent SRH issues.

**Study design:** the study adopted a cross-sectional design to study the state of knowledge, perceptions, and access of adolescents in Greater Banjul Area. This design was chosen because it gives a clear snapshot of the prevailing state of SRH knowledge, attitudes, and practices at one point in time, thus allowing identification of the factors that influence the SRH-related behaviours. The target population included adolescents aged between 15 and 19 years enrolled in grades 10 and 11. The inclusion criteria were that the participants had to fall within the age ranged and grade specifications and be available during the study. Students with special needs, such as hearing or speech problems, were excluded due to the lack of professional interpreters to facilitate communication. Two secondary schools in the Greater Banjul Area were selected through balloting based on their availability and willingness to participate in the study. A structured questionnaire was used for the data collection.

**Sample size determination:** the sample size for this study was determined using the Kish and Leslie (1965) method, which is designed to ensure statistical precision by calculating the minimum required number of participants. The study adopted a 95% confidence interval, a precision level of 5%, and an estimated population proportion of 0.5, which represents maximum variability. Based on these parameters, the minimum sample size was calculated to be approximately 385 participants. To account for a 10% non-response or attrition rate, the sample size was increased to 424 participants. Two secondary schools in the Greater Banjul Area were selected through a balloting process, ensuring that the schools were available and willing to participate in the study. The schools selected were Gambia Senior Secondary School in Banjul and SOS Hermann Gmeiner High School in the Kanifing Municipality. The total sample size of 424 participants was proportionally allocated between the two schools, based on their respective student populations. The distribution resulted in 227 participants from Gambia Senior Secondary School, which has a student population of 800, and 197 participants from SOS Hermann Gmeiner High School, with a student population of 700. This proportional allocation ensured that the sample was representative of the student populations at each school while accounting for the availability of students in both institutions.

**Sampling technique:** in this study, a multi-stage sampling technique was employed to ensure a representative selection of participants. In the first stage, one region was selected from the six administrative regions of Gambia (Greater Banjul Area, West Coast Region, Lower River Region, Central River Region, Upper River Region, and North Bank Region) through simple random sampling, and the Greater Banjul Area (GBA) was chosen through a balloting process. At the second stage, two towns within the GBA, the city of Banjul and the Kanifing Municipal Council (KMC), were selected using purposive sampling. Purposive sampling was employed at this stage to intentionally select towns (Banjul and KMC) within the Greater Banjul Area (GBA), as these towns have a higher concentration of secondary schools, ensuring accessibility and representativeness relevant to the study's objectives. Subsequently, one from a total of eligible secondary schools was selected from each town through balloting. Gambia Senior Secondary School was selected from Banjul, while SOS Hermann Gmeiner High School was selected from KMC. After the schools were selected, classrooms from grades 10 and 11 were targeted. Each school contained three grade 10 and three grade 11 classes, from which four classes per school were randomly selected through balloting. Finally, participants were recruited from the selected classes using simple random sampling, ensuring that only adolescents aged between 15 and 19 years were included in the study. This systematic and stepwise approach was designed to ensure a balanced and representative sample of adolescents from both towns, reflecting the diversity of the population within the Greater Banjul Area.

**Data collection:** data were collected using a structured questionnaire that was meticulously designed and pre-tested by the principal investigator. The questionnaire was divided into four sections: demographic information (age, gender, ethnicity, and family structure), knowledge of sexual and reproductive health rights (SRHR) topics such as contraception, STI prevention, and healthcare access, perceptions of SRH (attitudes, comfort levels discussing SRH, and perceived barriers), and access to SRH services (use and satisfaction with quality of care). Pre-testing was carried out with 50 students from a different school. The feedback from the pre-test was used to revise the questionnaire for clarity and appropriateness for the culture. Reliability was assessed using a test-retest method over a two-week interval, and a Kappa Cohen test (r=0.8) was obtained, indicating satisfactory reliability. Data collection was conducted over a two-month period among students in grades 10 and 11. The questionnaires were administered by three trained research assistants under the supervision of the principal investigator. Private classroom settings were used to ensure students had a secure and comfortable environment for answering sensitive questions. The study´s purpose was explained to the students, and questions were encouraged. Participation was voluntary, with students allowed to decline or withdraw at any time without consequences. Anonymity was maintained, and all responses were treated confidentially to uphold privacy.

**Data analysis:** data collected from the survey were documented, reviewed for accuracy, and entered into Microsoft Excel for cleaning. The IBM Statistical Package for the Social Sciences (SPSS) version 26 was used for data analysis. Descriptive analysis was performed to summarize all variables, with results presented as frequencies, percentages, means, and standard deviations. The dependent variable in this study was the level of knowledge on sexual and reproductive health rights (SRHR), categorized into low or high knowledge. Independent variables included demographic characteristics (age, gender, ethnicity, family structure), perceptions (perceived comfort and barriers), and access to SRH services. The mean knowledge score was calculated, and students scoring below the mean were classified as having low knowledge, while those scoring at or above the mean were categorized as having high knowledge. Chi-square tests were conducted to assess relationships between the dependent and independent variables. Logistic regression analysis was used to identify factors influencing SRHR knowledge and access. Knowledge scores were calculated by assigning 1 point for correct answers and 0 for incorrect answers. Perception responses were divided into perceived comfort and perceived barriers, with scores computed based on selected responses. Access responses were recorded as “accessible” and “not accessible,” and the mean score was used to classify students into good or poor access to SRH services. Statistical significance was set at p<0.05.

**Ethical considerations:** ethical approval was obtained from the University of Ibadan/University College Hospital (UCH) Ethical Committee (UI/EC/24/0385), and The Gambia Government and Medical Research Council Joint Ethical Review Committee (31125). Approval for conducting this research was further sought and received from SOS Hermann Gmeiner High School and Gambia Senior Secondary School (GSSS/P/001(014)). Written informed consent was obtained from those participants who were above 18 years, while for all participants below 18 years of age, informed consent of parents supported by participant assent was an obligatory requirement before inclusion in the study. They were assured that participation was entirely voluntary and that the information provided would be treated as strictly confidential. The ethical considerations that were followed in this study included respect for autonomy, the principle of beneficence, non-maleficence, and protection of data.

## Results

**Socio-demographic characteristics of adolescent students in the Greater Banjul Area (Gambia):** a total of 424 students participated in the study, with the majority aged 17 years (31.4%), followed by those aged 18 years (26.7%) and 16 years (19.3%) (Table 1). Most students were female (58.5%), while 39.9% were male, and a small percentage identified with a different gender orientation 7, 1.7%). Participants were evenly distributed across the two grades; 52.6% of the pupils were in grade 10, while 47.4% were in grade 11. By schools, 53.5% attended Gambia Senior Secondary School (Public) and 46.5% attended SOS Hermann Gmeiner high school (Private). Most of the participants identified as Gambians (90.1%), with 9.9% indicating a non-Gambian nationality (Table 1). The ethnic breakdown of the participants included: Mandinka 29.7%, Wolof 28.5%, Fula 19.1%, Jola 11.3%, and the rest belonged to other ethnic groups (Table 1). Family composition consisted of 46.2% participants who were in a nuclear family, 33.0% in an extended family, and 20.8% from a single-parent family. Parental education was reported as follows: 38.2% secondary school, 29.0% tertiary education, 20.8% with no formal education, and 12.0% with primary school. Most of the fathers were self-employed (46.7%) or employed (44.8%), while mothers were self-employed (41.7%) or employed (32.1%), with 26.2% being unemployed (Table 1).

**Table 1:** socio-demographic characteristics of grade 10 and 11 Students in Greater Banjul Area (Gambia) in 2024

Category	Subcategory	Frequency (%)
	15	36 (8.5%)
	16	82 (19.3%)
	17	133 (31.4%)
	18	113 (26.7%)
	19	60 (14.2%)
**Gender**	Male	169 (39.9%)
	Female	248 (58.5%)
	Gay	4 (0.9%)
	Lesbian	1 (0.2%)
	Bisexual	2 (0.5%)
Grades	10	223 (52.6%)
	11	201 (47.4%)
School	SOS Hermann Gmeiner high school	197 (46.5%)
	Gambia Senior Secondary School	227 (53.5%)
Nationality	Gambian	382 (90.1%)
	Non-Gambian	42 (9.9%)
Ethnicity	Mandinka	126 (29.7%)
	Wolof	121 (28.5%)
	Fula	81 (19.1%)
	Jola	48 (11.3%)
	Others	48 (11.3%)
Family type	Single parent	88 (20.8%)
	Nuclear	196 (46.2%)
	Extended	140 (33.0%)
Parent’s educational level	No formal education	88 (20.8%)
	Primary	51 (12.0%)
	Secondary	162 (38.2%)
	Tertiary	123 (29.0%)
Father’s occupation	Employed	190 (44.8%)
	Self-Employed	198 (46.7%)
	Not-Employed	36 (8.5%)
Mother’s occupation	Employed	136 (32.1%)
	Self-Employed	177 (41.7%)
	Not-Employed	111 (26.2%)

**Awareness and knowledge of SRHR among adolescents in the Greater Banjul Area (Gambia):** the findings revealed substantial gaps in students' awareness and understanding of sexual and reproductive health rights (SRHR). A total of 43.9% of students reported to have ever heard of SRHR, while 56.1% reported in the negative (Table 2). Only 12.7% of the students had attended training on SRHR, with the majority (87.3%) reporting no formal exposure. Knowledge of specific SRHR components was limited with 24.1% of the students identified the right to access contraception, 47.4% acknowledged the right to receive sex education, and 23.6% understood the right to consent to sexual activity. Furthermore, 43.4% of the students identified the right to access healthcare services, while 4.2% identified other rights. Schools were mentioned as the main source of information regarding SRHR by 54.0% of the respondents, followed by media (27.1%), friends (18.9%), family (17.2%) healthcare providers (13.4%), and community programs (10.1%). Some of the students (2.6%) reported other sources. Awareness of SRH services and laws was very low. Only 21.0% of the students reported knowing where to access SRH services, and 3.1% (n=13) reported knowing any SRH policies in The Gambia (Table 2). Knowledge levels were classified as good or poor based on a computed knowledge score, which was graded 0-3 as poor knowledge and 4-8 as good knowledge. A total of 21.9% of the students had good knowledge, whereas, 78.1% were classified as having poor knowledge.

**Table 2:** awareness, knowledge, and sources of information on sexual and reproductive health rights (SRHR) among students in the Greater Banjul (Gambia) Area in 2024

Question/Category			Yes n (%)	No n (%)
Ever heard of SRHR			186 (43.9%)	238 (56.1%)
Ever attended a training on SRHR			54 (12.7%)	370 (87.3%)
SRHR includes right to access contraception			102 (24.1%)	322 (75.9%)
SRHR includes right to receive sex education			201 (47.4%)	223 (52.6%)
SRHR includes right to consent to sexual activity			100 (23.6%)	324 (76.4%)
SRHR includes right to access healthcare services			184 (43.4%)	240 (56.6%)
SRHR includes other rights			18 (4.2%)	406 (95.8%)
Get information about SRHR from school			229 (54.0%)	195 (46.0%)
Get information about SRHR from friends			80 (18.9%)	344 (81.1%)
Get information about SRHR from family			73 (17.2%)	351 (82.8%)
Get information about SRHR from media			115 (27.1%)	309 (72.9%)
Get information about SRHR from healthcare providers			57 (13.4%)	367 (86.6%)
Get information about SRHR from community programs			43 (10.1%)	381 (89.9%)
Get information from other sources			11 (2.6%)	413 (97.4%)
Do you know where to access SRH services			89 (21.0%)	335 (79.0%)
Aware of any laws on SRH in the Gambia			13 (3.1%)	411 (96.9%)
**Statistical analysis**				
**Score Range**		**Knowledge Level**	**Count (%)**	
4-8	Good knowledge		93 (21.9%)	
0-3	Poor knowledge		331 (78.1%)	

**Perceptions and access to sexual and reproductive health rights (SRHR) services among adolescents in the Greater Banjul Area (Gambia):** the result on perceived comfort and attitudes towards access to sexual and reproductive health services indicated that 52.8% believed adolescents should receive SRH services, while 47.2% did not agree (Table 3). Comfort with discussing SRH issues varied highly depending on who was involved. Generally, students showed comfort with their friends in a discussion of SRH issues. The students, 39.6%, are very comfortable with their friends, while 25.9% would be somewhat comfortable. However, only 18.3% reported being very comfortable discussing SRH issues with teachers, and 27.8% were very comfortable speaking with healthcare providers. Discussions with parents were marked by discomfort, where 38.4% reported not being comfortable, while only 25.7% were very comfortable. Similarly, the community leaders were the least approachable, where 44.8% reported not being comfortable (Table 3). The perceived comfort was rated on a graded score. In this respect, the student respondents with positive perceived comfort comprised 46% and those with negative perceived comfort comprised 54%, as presented in Table 3. Moreover, the results from this study indicated that access to sexual and reproductive health services revealed that only 22.4% of the students reported ever accessing the SRH services, whereas the majority, about 77.6%, had not accessed them.

**Table 3:** adolescents' perceived comfort and access to sexual and reproductive health (SRH) services among secondary school students in the Greater Banjul Area (Gambia) in 2024

Adolescents’ perceived comfort				
**Question/Category**		**Subcategory**		**Frequency (%)**
Do you believe that adolescents should access SRH services?		Yes		224 (52.8%)
		No		200 (47.2%)
How comfortable are you in discussing SRH issues with parents?		Very comfortable		109 (25.7%)
		Somewhat comfortable		89 (21.0%)
		Not comfortable		163 (38.4%)
		Not applicable		63 (14.9%)
How comfortable are you in discussing SRH issues with friends?		Very comfortable		168 (39.6%)
		Somewhat comfortable		110 (25.9%)
		Not comfortable		99 (23.3%)
		Not applicable		47 (11.1%)
How comfortable are you in discussing SRH issues with teachers?		Very comfortable		69 (18.3%)
		Somewhat comfortable		107 (25.2%)
		Not comfortable		173 (40.8%)
		Not applicable		75 (17.7%)
How comfortable are you in discussing SRH issues with healthcare providers?		Very comfortable		118 (27.8%)
		Somewhat comfortable		86 (20.3%)
		Not comfortable		156 (36.8%)
		Not applicable		64 (15.1%)
How comfortable are you in discussing SRH issues with community leaders?		Very comfortable		59 (13.9%)
		Somewhat comfortable		97 (22.9%)
		Not comfortable		190 (44.8%)
		Not applicable		78 (18.4%)
**Perceived comfort level**				
Level		**Count (%)**	**Score range**	
Negative		229(54%)	0-2	
Positive		195 (46%)	3-5	
**Access to services**				
**Question/Category**		**Yes (%)**	**Non (%)**	**Na (%)**
Ever accessed SRH services		95 (22.4)	329 (77.6)	
Accessed at a public clinic/hospital		35 (8.3)	62 (14.6)	327 (77.1)
Accessed at a private clinic/hospital		32 (7.5)	65 (15.3)	327 (76.9)
Accessed at a community health centre		15 (3.5)	83 (19.6)	326 (76.9)
Accessed at a school clinic		14 (3.3)	84 (19.8)	326 (76.9)
Accessed at other places		3 (0.7)	92 (21.7)	329 (77.6)
Satisfied with services		64 (15.1)	29 (6.8)	331 (78.1)
Type of SRH service received, contraception		1 (0.2)	96 (22.6)	327 (77.1)
Type of SRH service received, HIV/STI testing		33 (7.8)	64 (15.1)	327 (77.1)
Type of SRH service received, sexual education/counselling		55 (13.0)	42 (9.9)	327 (77.1)
Type of SRH service received, prenatal care		2 (0.5)	94 (22.2)	328 (77.4)
Type of SRH service received, others		6 (1.4)	88 (20.8)	330 (77.8)
Ever accessed SRH services		95 (22.4)	329 (77.6)	
Accessed at a public clinic/hospital		35 (8.3)	62 (14.6)	327 (77.1)
**Level of access to services**				
**Score range**	**Level**	**Count (%)**		
0-2	Poor	330 (77.8%)		
3- 5	Good	94 (22.2%)		

**Similarly, the students who accessed SRH services reported utilizing the services in the following places:** public clinics or hospitals, 8.3%, and private clinics or Hospitals, 7.5%, respectively. community health centers (3.5%), school clinics (3.3%), and other places (0.7%) were less frequently utilized. Satisfaction with SRH services was reported by 15.1% of students, while 6.8% expressed dissatisfaction (Table 3). Regarding the type of SRH services received, HIV/STI testing (7.8%) and sexual education or counselling (13.0%) were the most frequently reported services. Contraception (0.2%), prenatal care (0.5%), and other SRH services (1.4%) were accessed less frequently. Analysis of access levels indicated that 22.2% of students had good access to SRH services, while 77.8% experienced poor access, with grade scores of 0-2 and 3-5 (Table 3). The study identified several key barriers that hinder adolescents´ access to sexual and reproductive health (SRH) services in the Greater Banjul Area. Cultural taboos (34.9%) and a lack of information (41.5%) were the most commonly reported obstacles. Other notable barriers included fear of judgment (22.9%), lack of services (22.2%), financial constraints (15.1%), distance to facilities (15.1%), and negative attitudes of healthcare providers (16.5%). Furthermore, 59.7% thought that SRHR education in schools was crucial and rated it as very important, while 19.6% of the participants rated it as important (Table 4). Despite the perceived value of SRHR education, students' opinions of the obstacles were generally negative; 90.8% of them acknowledged having perceived barrier. The score was computed as Yes (1-11) or No (0).

**Table 4:** barriers to accessing sexual and reproductive health services and the importance of sexual and reproductive health rights education among adolescents in the Greater Banjul Area (Gambia) in 2024

Barrier		Yes n (%)	No n (%)
Cultural taboos		148 (34.9%)	276 (65.1%)
Lack of information		176 (41.5%)	248 (58.5%)
Fear of judgment		97 (22.9%)	327 (77.1%)
Lack of services		94 (22.2%)	330 (77.8%)
Financial constraints		64 (15.1%)	361 (85.1%)
Distance to facilities		64 (15.1%)	360 (84.9%)
Attitudes of healthcare providers		70 (16.5%)	354 (83.5%)
Gender roles		57 (13.4%)	367 (86.6%)
Poverty		77 (18.2%)	347 (81.8%)
Peer influence		80 (18.9%)	344 (81.1%)
sexual and reproductive health inadequately covered in school curriculum		53 (12.5%)	371 (87.5%)
Other barriers		3 (0.7%)	421 (99.3%)
Importance of SRHR Education in school curriculum		Level of importance	Frequency (%)
		Very important	253 (59.7%)
		Important	83 (19.6%)
		Neutral	73 (17.2%)
		Not important	15 (3.5%)
Level of barrier perception			
Score range	Level	Count (%)	
0	No	39 (9.2%)	
1-11	Yes	385 (90.8%)	

**Statistical predictors of knowledge, perceived comfort, barriers, and access to sexual and reproductive health rights and services among adolescents in the Greater Banjul Area (Gambia):** the chi-square analysis revealed significant associations between specific variables and sexual and reproductive health (SRH) outcomes (Table 5). School affiliation was significantly associated with perceived barriers (p = 0.001), and access to SRH services (p < 0.001), indicating that school environments play a critical role in shaping students' SRH-related outcomes. Grade (p= 0.011), nationality (p= 0.034) and parent’s educational level (p = 0.030) were significantly associated with perceived comfort suggesting that perceived comfort in discussing SRH issues may vary with students' academic levels, nationality and parents’ educational level. No statistically significant associations were observed for gender, ethnicity, parental education, or parental occupation across SRH knowledge, perceived barriers, and access outcomes. The analysis indicated that school significantly influenced adolescents' knowledge on sexual and reproductive health rights. Students from Public School had an AOR of 0.587 (CI: 0.355 to 0.971, p = 0.038), indicating a significant association between school and knowledge. Age, grade, school, nationality, family type and parents’ educational level were found to be predictors of perceived comfort in discussing SRH issues (Table 6).

**Table 5:** chi-square analysis of determinants for SRH knowledge, perceived barriers, and access among secondary school students in the Greater Banjul Area, The Gambia, 2024

Variable	Knowledge		Perceived comfort		Perceived barriers		Access	
	c2	P-value	c2	P-value	c2	P-value	c2	P-value
Age	7.648	0.105	0.459	0.979	5.660	0.224	4.974	0.315
Gender	5.203	0.077	0.929	0.671	4.736	0.121	1.769	0.369
Grades	2.047	0.160	6.880	0.011*	2.306	0.134	0.210	0.727
School	3.360	0.078	3.519	0.064	11.627	0.001*	19.400	<0.001*
Nationality	0.227	0.844	4.753	0.034*	1.098	0.405	1.959	0.174
Ethnicity	4.023	0.409	8.533	0.074	5.887	0.208	4.039	0.391
Family type	2.604	0.269	5.633	0.060	1.260	0.522	4.876	0.092
Parent’s education	6.189	0.103	8.978	0.030*	4.515	0.243	3.215	0.348
Father’s occupation	1.987	0.350	1.877	0.388	4.959	0.123	0.153	0.894
Mother’s occupation	0.009	1.000	3.508	0.173	0.405	0.808	1.949	0.387

_* = Statistically significant results (p < 0.05); SRH:sexual and reproductive health

**Table 6:** logistic regression analysis of predictors associated with adolescents' perceived barriers and access to sexual and reproductive health rights and services in the Greater Banjul Area, The Gambia, 2024

Variable		AOR	Confidence interval		P-value
			Lower limit	Upper limit	
**Predictors of adolescents’ perceived comfort on sexual and reproductive health**					
Age	19	1	-	-	-
	15	0.372	0.145	0.955	0.040*
	16	0.541	0.252	1.162	0.115
	17	0.648	0.328	1.283	0.213
	18	0.891	0.458	1.733	0.734
Grade	10	1	-	-	-
	11	0.500	0.317	0.789	0.003*
Nationality	Gambian	1	-	-	-
	Non-Gambian	2.888	1.377	6.057	0.005*
School	Private	1	-	-	-
	Public	1.866	1.214	2.867	0.004*
Parent’s educational level	Tertiary	1	-	-	-
	No formal education	0.609	0.335	1.106	0.103
	Primary	0.815	0.407	`1.633	0.563
	Secondary	0.485	0.290	0.812	0.006*
Family type	Single- parent	1	-	-	-
	Extended	0.555	0.315	0.978	0.042*
	Nuclear	0.786	0.459	1.346	0.380
**Predictors of adolescents’ perceived barrier on sexual and reproductive health**					
Age	19	1	-	-	-
	15	1.272	0.266	6.082	0.763
	16	1.997	0.574	6.947	0.277
	17	1.996	0.718	5.550	0.185
	18	3.403	1.147	10.096	0.027*
School	Private	1	-	-	-
	Public	0.253	0.107	0.601	0.002*
**Predictors of adolescents’ access to sexual and reproductive health services**					
Age	19	1	-	-	-
	15	1.337	0.416	4.293	0.626
	16	1.812	0.706	4.650	0.216
	17	1.796	0.748	4.310	0.190
	18	2.702	1.167	6.252	0.020*
School	Private	1	-	-	-
	Public	0.320	0.190	0.539	<0.001*
Family type	Single-parent	1	-	-	-
	Extended	0.658	0.347	1.247	0.199
	Nuclear	0.489	0.261	0.916	0.025*

_* = Statistically significant results (p < 0.05)

The logistic regression analysis identified the main factors that affected adolescents' perceived barriers and access to sexual and reproductive health rights and services in the Greater Banjul Area. Among the perceived barriers, Age category 15, 17 and 18 years were 27%, 99%, 40% respectively, more likely to report perceived barrier compared to those who were 19 years old (RC) (AOR=1.272, 95% CI; 0.266-6.082, p 0.763), (RC) (AOR=1.996, 95% CI; 0.718-5.550, p 0.185) and (AOR=3.403, 95% CI; 1.147-10.096, p 0.027) respectively. This suggests the odds of perceiving barriers among individuals aged 15 years were approximately 27% higher compared to those aged 19 years, whereas school attendance odds of perceiving barriers among respondents in public school are 25% lower compared to those in private school (RC) (AOR=0.253, 95% CI; 0.107-0.601, p = 0.002), this suggests that individuals in the former group typically perceive fewer barriers than those in the latter group (Table 6). For access to SRH services, the tendency of accessing SRH services for respondents aged 18 years are about 70% higher compared to those in the reference group (RC) (AOR=2.702, 95% CI; 1.167-6.252, p 0.020). This indicates that those in this age category have significantly more access to SRH services than their 19-year-old counterparts. Attending a public school was significantly associated with low access to SRH services (AOR = 0.320, 95% CI: 0.190-0.539, p < 0.001). The odds of accessing SRH services for participants from nuclear families are approximately 49% lower compared to those from single-parent families (RC) (AOR=0.489, 95% CI; 0.261-0.916, p 0.025). This shows that individuals from nuclear family structures have poor access to SRH services relative to those in single-parent families.

## Discussion

The study evaluated adolescents´ knowledge, perceived comfort, barriers, and access to sexual and reproductive health (SRH) services in The Gambia. The findings revealed significant gaps in SRH knowledge, low comfort levels in discussing SRH issues, and substantial barriers to accessing services, reflecting universal and context-specific challenges faced by adolescents in low-resource settings. Adolescents' knowledge of SRH was low, with only 43.9% reporting awareness and 12.7% attending SRH training. These findings are consistent with the study by Bolakotagi and Latif [15], which highlighted a low level of SRH awareness among adolescents, with a small percentage reporting awareness and attending SRH training. Similarly, Ilori *et al*. [16] found that just 13.1% of adolescents were aware of SRH services, with higher awareness in urban areas in Oyo state (Nigeria). In contrast, studies conducted in Nepal [17] and Northwest [18] Ethiopia revealed significantly higher levels of SRH knowledge among adolescents. Moreover, the low percentage (21%) of Gambian adolescents aware of SRH service access mirrors findings from Onitsha, Nigeria, where adolescents demonstrated limited awareness, particularly regarding contraception and fertility awareness [19]. Usonwu *et al*. [20] emphasized that in sub-Saharan Africa, limited parental communication on SRH and reliance on cultural and religious norms hinder adolescents' access to accurate information, a factor that appears consistent with the Gambian context. Moreover, the absence of SRH clubs and structured curricula in The Gambia may explain these deficits, as highlighted in studies from Nigeria and Kenya, where such initiatives significantly improved knowledge [21,22].

Private school students in The Gambia demonstrated significantly higher SRH knowledge than their public school counterparts. This trend aligns with findings from Nigeria, where private school adolescents benefited from smaller class sizes and better-resourced curricula that emphasized SRH awareness [19]. The inequities in SRH education between public and private schools, as noted by Usonwu *et al*. [20], underscore systemic disparities in resource allocation that affect adolescents' access to critical health information. Comfort levels in discussing SRH issues were notably low, with only 46% of adolescents expressing comfort. Discussions were primarily with friends, followed by healthcare providers and parents. This aligns with findings from Tanzania and Nigeria, where cultural norms and societal taboos hindered open conversations, particularly with parents [19,23]. The study revealed that Gambian adolescents were less comfortable discussing SRH compared to non-Gambians. Similar findings were reported in Kenya, where cultural restrictions shaped adolescents' willingness to discuss SRH topics with adults [22]. Adolescents whose parents had lower educational level in this study were less comfortable discussing SRH (AOR=0.485, 95% CI: 0.290-0.812, p=0.006). This is similar with findings from Ethiopia and Nigeria, where higher parental education positively influenced SRH communication [24,25]. The similarities may stem from similar culture in African societies, where parental roles in SRH communication may be the same in sub-Saharan African countries [20]. The findings from this study show that adolescents attending government schools are more likely to perceive comfort in discussing SRH issues compared to those in private schools, a contrasting finding was revealed in a similar study in Nigeria where students in private schools were found to be 3 times more comfortable in discussing SRH issues compared to those the government schools [26]. With grade adolescents in higher grades are found to be 50% less likely to be comfortable in discussing SRH issues compared to those in lower grades. In a similar study conducted in Northern Ethiopia adolescents in lower grades were 2.3 times and 2.2 times more likely to discuss SRH issues with their parents compared to those in higher grades [27].

Barriers to accessing SRH services were predominantly attributed to lack of information (41.5%) and cultural taboos (34.9%), findings consistent with research in Kenya and Ethiopia that identified trust in services, confidentiality concerns, and fear of judgment as significant barriers [22,28]. However, financial constraints, often a key barrier in other contexts, were less emphasized in this study, possibly reflecting differences in healthcare systems or economic factors. Usonwu *et al*. [20] highlighted those cultural and religious norms in sub-Saharan Africa heavily influence adolescents' ability to seek SRH services, which resonates with the findings in this study. Private school students reported significantly fewer barriers than their public school counterparts, similar to observations in Nigeria and Kenya, where private school students had better access to SRH services [21,22]. Access to SRH services was notably limited, with only 22.4% of adolescents utilizing services, primarily at public clinics, and just 15% reporting satisfaction. HIV/STI testing and sexual education counselling were the most accessed services, aligning with findings from Ethiopia and Ghana but with lower utilization rates in Gambia [23,29]. Adolescents in private schools were significantly more likely to access these services, emphasizing systemic inequities. Expanding school-based SRH programs and youth-friendly services is essential to bridging these gaps [20].

**Limitation of the study:** the study was conducted only in the Greater Banjul Area and is, therefore, less representative of adolescents in other regions, especially rural regions where cultural norms and access to services differ. Reliance on self-reported data could have introduced biases, as participants may provide information that is not accurate or that is socially desirable, especially on sensitive topics like sexual and reproductive health. The results are further confined to the responses of school-going youth, excluding out-of-school adolescents and bypassing the problems besetting those who are not enrolled in formal education.

## Conclusion

This study has brought to light the wide gaps in the status of knowledge, perception, and access to SRH services among adolescents in the Greater Banjul Area. The findings revealed that most adolescents lacked awareness about their rights regarding SRH issues, experienced low comfort in discussing these issues, and faced barriers involving cultural taboos, inadequate information, lack of youth-friendly services, fear of judgment, and negative healthcare provider attitudes. Factors such as age, grade level, school type, family structure, and parental education were found to influence adolescents´ SRH knowledge, perceptions, and access. Insufficient SRH education and disparities between private and public school students further exacerbate these challenges. The findings emphasize the need for integrating evidence-based, age-appropriate SRH education into national curricula, addressing these disparities, and establishing accessible, youth-friendly health services. Community engagement to reduce cultural barriers and improve healthcare provider attitudes is critical to ensuring equitable adolescent health outcomes.

### What is known about this topic

- Adolescents in sub-Saharan Africa face barriers to accessing sexual and reproductive health (SRH) services, including cultural taboos, stigma, and lack of youth-friendly facilities;
- Knowledge of SRH rights, such as contraception and STI prevention, is limited due to inadequate education and restricted access to information;
- Sociocultural norms often prevent open discussions about SRH, particularly with parents and community leaders.

### What this study adds

- Reveals that only 43.9% of adolescents in the Greater Banjul Area were aware of their SRH rights, with 12.7% having received formal SRH training;
- Identifies key barriers such as barriers to access services, including lack of information (41.5%) and cultural taboos (34.9%) affecting access to SRH services;
- Highlights the influence of factors like age, school type, grade level, and parental education on adolescents' SRH awareness and access.
